# Growing up without violence (GWV): Results of a cluster randomised trial of a school-based intervention preventing adolescent sexual abuse and exploitation in Brazil

**DOI:** 10.1371/journal.pone.0342274

**Published:** 2026-07-30

**Authors:** Ligia Kiss, Marto Leal, Ana Paula Portella, Heloína Paiva, Agnese Iuliano, Malini Pires, Helen Shipman, Elizabeth Anderson, Yuki Lo, Ligia Kerr, Carl Kendall, Dario Brito Jr, Marina Barros, Baptiste Leurent

**Affiliations:** 1 Associate Professor in Social Epidemiology, Institute for Global Health, University College London, London, United Kingdom; 2 Fundação Oswaldo Cruz Ceará, Fortaleza, Brazil; 3 Research Coordinator, consultant, Department of Technology and Communication, Universidade Católica de Pernambuco, Recife, Brazil; 4 Research Fellow, consultant, Universidade Católica de Pernambuco, Recife, Brazil; 5 Research Fellow, Institute for Global Health, University College London, London, United Kingdom; 6 Research Fellow, consultant, Institute for Global Health, University College London, London, United Kingdom; 7 Research Consultant, Research and Evaluation, Freedom Fund, London, United Kingdom; 8 Head of Research and Evaluation, Research and Evaluation, Freedom Fund, London, United Kingdom; 9 Professor of Epidemiology, Universidade Federal do Ceará, Fortaleza, Brazil; 10 Visiting Professor of Medical Anthropology and Community Health, Universidade Federal do Ceará, Fortaleza, Brazil; 11 Assistant Professor, Universidade Católica de Pernambuco, Recife, Brazil; 12 Assistant Professor, Universidade Católica de Pernambuco, Recife, Brazil; 13 Associate Professor in Medical Statistics at the Department of Statistical Science, University College London, London, United Kingdom; IFPRI: International Food Policy Research Institute, UNITED STATES OF AMERICA

## Abstract

**Introduction:**

Child sexual abuse and exploitation (CSAE) are pervasive problems that significantly affect the health of children and adolescents. This paper presents the results of a cluster randomised controlled trial (cRCT) evaluating a school-based intervention designed to prevent CSAE in Brazil through education.

**Methods:**

We conducted a two-arm cRCT with parallel assignment in two Brazilian municipalities. Sixty schools were randomly allocated to intervention and control arms (30 per group) using stratified randomisation based on municipality and school type (municipal and state-run). We invited 50 randomly selected students, aged 12–17, from each school to complete surveys at baseline and endline. Teachers enrolled in GWV training completed an implementation questionnaire. Our primary analysis focused on a cross-sectional comparison of students’ CSAE risk knowledge between control and intervention schools.

**Results:**

We found no statistically significant difference in adolescents’ CSAE risk knowledge between the study arms (adjusted mean difference: 0.02, 95% CI −0.19 to 0.23, p = 0.87). There was borderline evidence of interaction by gender, with a greater difference in knowledge scores among girls (p = 0.032). No significant association was found between the number of GWV components implemented and knowledge scores.

**Discussion:**

Several factors may contribute to this result: the intervention might not have been implemented with sufficient fidelity or intensity, the true effect of GWV could be minimal and below the study's detection threshold, the intervention may have influenced outcomes that were not measured, or the intervention may simply lack effectiveness. Further rigorous evaluations and implementation studies are essential to ensure that resources are directed toward effective strategies.

**Trial registration:**

The American Economic Association’s registry for randomised controlled trials (AEA RCT Registry). RCT ID: AEARCTR-0011299, https://www.socialscienceregistry.org/trials/11299

## Introduction

Child sexual abuse and exploitation (CSAE) are associated with severe negative health impacts, including early pregnancy, sexually transmitted infections (STIs), gynaecological problems, and violence-related injuries. The mental health consequences of CSAE can be profound and long-lasting, and include anxiety, depression, post-traumatic stress disorder, developmental problems, suicidal behaviour, self-harm, and substance misuse [1–3]. Many of these problems can persist into adulthood. Nonetheless, despite adolescents’ high risk of sexual abuse and exploitation compared to other age groups, this public health agenda remains underdeveloped across the world [3].

The World Health Organisation (WHO) defines child sexual abuse as actual or threatened physical intrusion of a sexual nature, whether by force or under conditions of inequality or coercion. Sexual exploitation involves the abuse of a position of vulnerability, power, or trust, for sexual purposes [4]. Child sexual exploitation encompasses any form of transactional sex, including sex trafficking, the sale of images or videos depicting the sexual assault of children, sex tourism, and paid sexual performances such as livestreaming [5].

The global prevalence of sexual violence against children is estimated at 18·9% (95% uncertainty interval [UI] 16·0–25·2) for females and 14·8% (9·5–23·5) for males in 2023 [6]. A recent study involving a national sample of Brazilian students aged 13–17 reported prevalence rates of 14.6% for child sexual abuse and 6.3% for rape [7]. Research in the Recife Metropolitan Region (RMR), our study site, estimates that 9.62% (5.44–18.83%) of girls under 18 were commercially sexually exploited between 2019 and 2022. Of these adolescents, 78.6% were still enrolled in school when first exploited [8,9].

Evidence suggests that the health burden of CSAE can be prevented through targeted investments in public health approaches that emphasise education and awareness, early intervention, and adolescent-led responses [3,10]. A meta-analysis of 24 studies found that school-based education programs aimed at addressing sexual abuse can enhance children's knowledge of CSAE, improve self-protective behaviours, increase the likelihood of disclosure, and reduce feelings of self-blame. However, there is limited evidence regarding the effectiveness of these interventions in low- and middle-income countries [11].

For more than two decades, Brazil has been promoting policies to address sexual violence and sexual exploitation of children and adolescents [12]. The country is equipped with local and regional social service offices to address social risks and rights violations, including violence against children [13]. Although these resources exist, schools often lack the protocols, training, and support to detect, manage, and refer cases of CSAE [14–16]. Growing-up Without Violence (GWV) aims to address this gap through a school-based curriculum to reduce students’ risks of sexual abuse and exploitation and increase their reporting skills. By providing schools with structured capacity-building sessions for teachers, evidence-informed curriculum materials, and practical guidance on identifying, responding to, and referring cases of abuse, GWV equips institutions with trained personnel, frameworks and communication material to deliver CSAE prevention education.

GWV is a large-scale intervention in Brazil aimed at preventing CSAE. The intervention was developed and is implemented by Canal Futura, a non-profit arm of the Roberto Marinho Foundation, the largest media conglomerate in Brazil. GWV was initially developed in 2009 through thematic fora and consultations with experts and adolescent CSAE survivors. These fora periodically revise strategies, informing priorities and best implementation practices, and contributing to content development. The intervention's curriculum aims to enhance students’ understanding of CSAE risks, self-protection strategies, and reporting mechanisms, while also equipping teachers with the skills needed to identify and respond effectively to cases. GWV assumes that, with this knowledge, adolescents will be better able to recognise and respond effectively to CSAE risks. The intervention targets students aged 7 and older enrolled in public schools in Brazil and is tailored to different age groups and their developmental stages.

To implement the intervention, Canal Futura leverages its partnerships with Brazilian local authorities, who identify the target schools that will receive the intervention. Typically, these are public schools managed by these authorities at both the municipal and state levels. These sessions focus on themes presented in the intervention's audiovisual content, including family-based child sexual abuse, sexual exploitation, victim identification, active listening, reporting CSAE, intersectoral victim support, children's emotional health (including self-harm risks), and discrimination related to gender identity and sexual orientation. Supplementary Material (S1 Table) provides a summary of the intervention’s content, target population, and materials.

All GWV trainers have a professional background in education and sexual violence prevention and undergo intensive training on the intervention. This training begins with an induction meeting, followed by five two-hour capacity-building sessions in which Canal Futura enhances participants’ knowledge about CSAE and provides support for planning and implementing GWV resources in school classrooms. During the planning, implementation, and feedback stages, trainees receive ongoing support and share their experiences through a social media group managed by Canal Futura. School staff ultimately make the final decisions on GWV content delivery and frequency, based on their priorities, capacity, and available resources. In the final training session, participants engage in a workshop to share their experiences with the curriculum and discuss long-term plans for project sustainability.

This paper presents findings from the Growing Up Without Violence (GWV) trial, which evaluated the effectiveness of the GWV curriculums a school-based prevention program for child sexual abuse and exploitation (CSAE). Our study aimed to assess the intervention's impact on students’ knowledge of the risks associated with sexual abuse and exploitation, as well as their self-protective behaviours.

## Materials and methods

### Study design

The GWV Trial is a two-arm parallel repeated cross-sectional Cluster Randomised Controlled Trial (cRCT) of the GWV curriculum, a school-based complex social intervention to prevent CSAE. This evaluation design was employed for three reasons. First, it avoids the attrition that would arise from following individual students over time in a context with high school absenteeism. Second, it reflects the population-level estimand of interest (the effect of GWV on knowledge levels across the school population at a given point in time), rather than individual trajectories of change. Third, it is consistent with the pragmatic, school-level nature of the intervention, in which the unit of delivery and randomisation is the school rather than the individual student.

### Patient and public involvement

The study was planned and conducted in collaboration with local authorities, school managers, and teachers, who were initially engaged in presentations and discussions in Cabo de Santo Agostinho in 2023. Many of the authors of the manuscript were present during this event. Subsequent meetings were held online. Research questions and study outcomes were co-developed with Canal Futura and the Freedom Fund, drawing on their experience with schools and staff training. School managers played a crucial role in obtaining consent from parents. The methods for the study were specified in the research call by IPA. Members of the public were not involved in determining the plans for disseminating the study.

### Schools and participant inclusion criteria and selection

Public schools in the municipalities of Cabo de Santo Agostinho and Jaboatão dos Guareligarapes (hereafter referred to as Cabo and Jaboatão, respectively), with students aged 12–17, served as the units of randomization for the cRCT. Both municipalities are located in the Metropolitan Region of Recife, in the northeastern Brazilian state of Pernambuco, and are recognized as hotspots for adolescent sexual exploitation in Brazil [17]. Cabo is home to the largest port in Brazil Northeast, the Suape Port. As a transit point for sexual tourism, the municipality experiences high rates of adolescent sexual exploitation and child drug use [18]. Jaboatão is one of the three municipalities with the highest population density in Pernambuco and is also recognized for having a high incidence of adolescent sexual exploitation [19].

All schools partaking in the study were publicly funded and followed the normative national educational curricula. From each selected school, two independent cross-sectional samples of students were invited to participate in the baseline and endline surveys. Eligible students were those in the final two years of primary/elementary school or in secondary school (ages 12–17) who could understand the survey questions and whose parents did not withdraw consent for their participation in the survey.

### Randomisation and masking

The trial statistician prepared a randomisation program using a random number generator in Stata [20]. Allocation between the two trial arms was with a 1:1 ratio stratified by municipality and school type, composing three strata: i) Cabo, municipal schools, ii) Cabo, state schools, and iii) Jaboatão, state schools. The trial coordinator ran the randomisation program and identified which of the 60 schools were allocated to the GWV intervention or waitlist control.

The trial coordinator informed Canal Futura of the allocation. Schools and students were not masked to the allocation but were not informed until after the baseline survey (described below). The principal investigator, the trial statistician, and the research team were blinded to study allocation. Unmasking occurred once all the endline data were collected and processed, and the statistical analysis plan was approved and published. Implementation data were processed after unmasking.

### Intervention and control

After being informed about the schools randomised to the GWV arm, Canal Futura approached them to schedule the training sessions.

Schools in the control arm did not receive the intervention and adhered to standard national guidelines, which typically require schools to implement activities focused on the week of May 18th, the Brazilian National Day to Combat Abuse and Sexual Exploitation of Children and Adolescents. Government guidelines mandate that Brazilian schools promote activities during this week to raise awareness about CSAE. However, these schools do not receive any training or guidance on the content or implementation of educational activities.

Each intervention school selected two teachers to receive training. Participating teachers attended an induction meeting where they were familiarised GWV's aims, principles, and curriculum content. The selected teachers were trained on the GWV material, following Canal Futura's standard methodology, as previously described. GWV does not use an explicit cascade model in which trained teachers subsequently train their peers, although informal transmission of training content between educators may occur and influence practice within schools. To ensure that children in the control arm also received the information covered by the GWV intervention, Canal Futura offered the intervention to schools in the control arm after the study's endline.

As with most complex social interventions implemented at scale, some heterogeneity in delivery across schools was expected, reflecting real-world implementation conditions, consistent with the pragmatic nature of this trial.

### Data collection

Students from the participating schools were invited to complete a survey at baseline in May 2024 (just after randomisation, and before the intervention was implemented), and at endline in September (approximately 4 months after baseline).

For both surveys, students were randomly selected from lists of all eligible participants at each school. The average number of eligible students per school was 273, ranging from 27 to 690. We piloted the survey in one municipal school in Cabo and one state school in Jaboatão. Given the high levels of student absenteeism observed in the baseline pilot results (approximately 28% of students in the selected sample), we randomly selected 70 students from schools with larger enrolments (n > 70), intending to interview 50.. For the four schools whose total eligible enrolment was fewer than 50 students, all eligible students were invited to participate.

School managers supported the research team in implementing an opt-out informed consent process with all parents of selected students. Parents were provided with information about the study and given the opportunity to withdraw their child’s participation. School managers contacted eligible parents through their usual communication channels (primarily WhatsApp groups), where they shared study information, responded to queries with support from the research team, and offered the option to opt out. Lists of parents who declined participation were subsequently shared with the research team, and the corresponding students were removed from the sampling frame.

Participating students received an electronic information sheet on the survey tablets explaining that the study included questions about potentially distressing past experiences and advising them not to participate if they believed this could cause discomfort. At both baseline and endline, fieldwork supervisors briefed students on the study procedures and ethical safeguards, emphasising voluntary participation, the right to refuse or withdraw, and the confidentiality and anonymity protections. The survey tablet was programmed to allow access to the questionnaire only after students provided active consent.

Fieldwork supervisors and researchers were trained using an adapted version of the WHO ethics protocols for research on violence against women [21] and on human trafficking [21], as well as guidelines for ethical research with children [22]. Training covered interviewing procedures, safety and confidentiality, responding to sample, referral pathways, and safeguarding of both participants and research staff. A specialised psychologist conducted an additional session on responding to children who exhibited distress signs or required immediate assistance.

Students completed self-administered surveys lasting 30–45 minutes. Fieldwork researchers ensured privacy, assisted with understanding the content, and provided individual support before and after the survey (maintaining a ratio of one researcher to five participants). The research team ensured that interviews were conducted in a private and safe environment, that participants fully understood the consent procedures, and that they freely agreed to participate and provided voluntary written assent. Details of the trial's ethical procedures can be found in the protocol paper [23].

Teachers who participated in the GWV training were also invited to fill in a survey about their implementation of the GWV curriculum. Two staff members in the trial fieldwork team implemented this survey immediately before the endline. Researchers sent the monitoring questionnaire to teachers in intervention schools through email and phone. They followed up by phone with those who did not initially respond.

Ethical approval for this research was granted by the Research Ethics Committee at the University College London (Project ID/Title: 24085/001), and the Brazilian National Research Ethics Committee (CAAE: 66827122.0.0000.5206).

### Outcomes

The primary outcome of the cRCT was children's knowledge of inappropriate sexual advances and acts, including grooming. We utilized the revised Children’s Knowledge of Abuse Questionnaire (CKAQ-R), which has been validated in Portuguese [24], to assess children's understanding of sexual abuse prevention concepts at both baseline and endline [25]. Children responded to 12 items, indicating whether they agreed, disagreed, or were unsure if the described behaviour was abusive. We scored the responses based on evidence-based guidelines and definitions of abuse. Correct identifications of abusive behaviour received 1 point, while incorrect responses (i.e., failing to identify abuse or mislabelling appropriate behaviour as abuse) and “unsure” responses received 0 points. The total CKAQ-R score was derived by summing all responses, yielding a range of 0–12, with higher scores indicating greater knowledge of abuse.

Additionally, we measured the effect of GWV on three secondary outcomes: i. knowledge of risks associated with online sex; ii. intention to seek help in cases of sexual abuse or exploitation, and iii. recall of recent exposure to school-based CSAE prevention activities. Knowledge of risks associated with online sex was measured by fifteen questions adapted to themes explored in the GWV curriculum, such as sexting, cyber grooming, and online sexual exploitation. Correct answers were scored as 1 point, and incorrect and “unsure” responses as 0 points. The total knowledge score was derived by summing all questions, with higher scores indicating greater knowledge (range 0–15). We assessed intention to seek help in cases of sexual abuse or exploitation through positive responses to the question “Would you seek help in a bad situation where you had a nude leaked or had your intimacy exposed or were sexually harassed online?.” This outcome was scored 1 if the student answered “yes” and as 0 if they answered “no” or “unsure”. Recall of recent exposure to school-based CSAE prevention activities was determined by responses to the question “Have you ever participated in teaching activities, workshops, or classes on how to prevent or react to situations in which someone wants to touch your body in a way that bothers you or that you don't want?”. This outcome was scored as 1 if the student answered “yes” and as 0 if they answered “no” or “unsure”. Further details on the outcomes’ definitions are available in the published protocol [26] and the Statistical Analysis Plans [26].

### Other measures

Other measures captured during baseline and endline included students’ characteristics, such as age, gender, and their experience of sexual abuse and exploitation. Items on sexual exploitation were based on the items used in the Violence Against Children (VAC) and Youth Surveys [27] and research by Dunkle, Jewkes [28]. These items were refined through recommendations by Wamoyi, Ranganathan [29]. We measured both transactional sex and sex work. We used questions from an adapted version of the questionnaire for the Project Sao Paulo for the social development of children and adolescents (SP-PROSO) [30].

Data on intervention implementation included the number of teachers registered to attend the GWV trainings and data from the teachers’ survey, including how many ‘components’ (i.e., specific material provided by GWV or topics covered as part of the training) they had already used in class. We used the number of components to define a level of GWV implementation (dose). We categorised schools into three groups: no data (school did not implement the intervention, or no teachers completed the implementation survey); lower implementation (reported average components used below or equal to 3); higher implementation (average components used above 3). We considered components implemented through the reported use of specific programme guidelines and learning materials for student activities (e.g., educational GWV kit, audiovisual series, workbooks on GWV themes, campaign materials, and case studies).

### Sample size

The study was powered to detect a small to medium standardised difference of 0.25 on the CKAQ-R at the endline. The sample size was calculated using Stata [20]. Assuming an intra-cluster correlation coefficient (ICC) of 0.07, 60 schools (30 per arm) with 50 students sampled in each school (corresponding to a design effect of 4.43 [31] provide 90% power to detect an effect size of 0.25 at the 2-sided alpha level of 5%. The observed ICC in the trial data was 0.06, close to the assumed value of 0.07, confirming that the sample was adequately powered.

### Statistical analysis

Details of the statistical analysis methods are reported in the Statistical Analysis Plan [26].

Descriptive statistics of schools and students’ characteristics at baseline and endline are reported. The primary estimand of interest is the average difference in CKAQ-R score at endline between schools randomised to receive the GWV curriculum compared to schools randomised to follow the standard curriculum. We compared the primary outcome (CKAQ-R score at endline) between arms using a cluster-level summary approach to take into account the clustering. We derived the average CKAQ-R score for each school, which were then compared between groups using a linear regression to estimate the mean difference between groups [32,33]. This approach was chosen given its appropriate control of type I error rate, even in studies with few clusters [34]. An equal weight was given to each school, which corresponds to estimating the average intervention effect expected for a school rather than a student-level average. In this sampling design, approximately equal numbers of students were selected per school (target of 50), meaning school-level means were estimated with similar precision across schools regardless of the variation in total school enrolment. We adjusted the analysis by the schools’ mean CKAQ-R score at baseline and stratified by randomisation strata. We compared the continuous secondary outcome (knowledge of risks associated with online sex) similarly, adjusting for the equivalent outcome at baseline. For the binary outcomes (seeking help in case of online abuse and participation in sexual abuse prevention activity), we reported both the risk difference and risk ratio, estimated using a cluster-level approach stratified by randomisation strata. We conducted the hypothesis tests based on the risk differences. The risk ratios were based on the ratio of geometric means, as described in Hayes [32] and Thompson et al [33]. The ratio of geometric means was used as the cluster-level analogue of the risk ratio, consistent with the school-level summary approach adopted throughout the analysis.

We conducted a comparative analysis according to the randomisation arm, regardless of the intervention received (intention-to-treat). The analysis, therefore, included the three schools in the GWV arm whose teachers did not attend the trainings. As the trial used a repeated cross-sectional design with independent samples of students surveyed at baseline and endline, individual-level longitudinal follow-up was not possible. This intention-to-treat estimand reflects the average effect of being offered the GWV programme under real-world conditions, including variations in uptake and delivery heterogeneity inherent in a pragmatic trial of a complex social intervention.

We then estimated the effect modification between students’ age and gender on the primary outcome, using linear mixed models with an interaction term between arm and the baseline covariates (age or gender). The models included a random-effect by school and were adjusted by baseline mean CKAQ-R and randomisation strata. We used a different analytical approach than the primary analysis to facilitate estimation of the participant level interaction.

Finally, we conducted a pre-specified exploratory analysis examining the association between the intervention ‘dose’ and the primary outcome in the schools randomised to the intervention. We categorised the schools into three groups based on teachers’ responses to the implementation survey (see ‘other measures’) and performed an overall test for differences using linear regression. This analysis compared the school-level data (mean endline CKAQ-R score), adjusting for strata and baseline CKAQ-R score. Because some schools in the intervention arm did not receive the training, we also conducted a post-hoc analysis aiming to estimate the causal effect in complying schools using instrumental variable methods [35]. A two-stage least squares estimation was used, based on the adjusted cluster-level residuals of the primary outcome, using having received the GWV training as endogenous variable and the randomisation as an instrument. It assumes that the randomisation had no effect on the outcomes for the schools that did not receive the training.

We conducted all analyses in Stata v18 [20] and used the clan command for cluster-level analysis [33].

## Results

### Recruitment and randomisation

**Fig 1:**
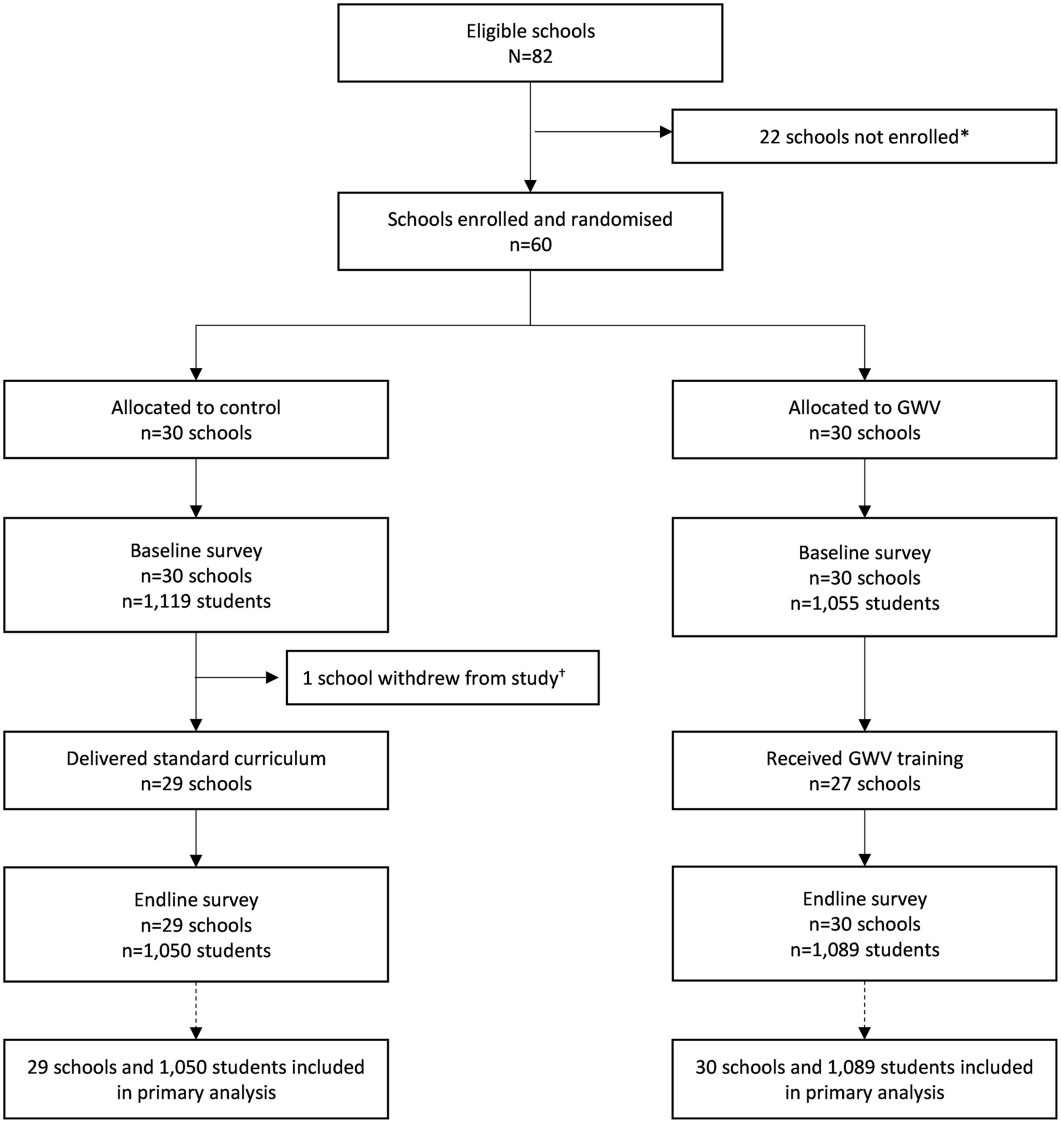
Trial flow chart.

We identified 82 public schools with students aged 12–17 in Cabo and Jaboatão municipalities (Fig 1). We enrolled all 31 municipal schools from Cabo in the trial, along with 29 randomly selected state schools from both Cabo and Jaboatão. All 60 schools were randomized, with half (n = 30) allocated to receive the GWV training and the other half (n = 30) assigned to the control arm. Fig 1 shows the trial flow chart.

### Baseline

We conducted the baseline survey between April and May 2024 with 2,174 students (36.2 students per school) (Table 1).

**Table 1:** Baseline characteristics.

	Control	GWV	All
**School characteristics**			
**Number of schools**	30	30	60
**Strata**			
Cabo de Santo Agostinho – Municipal schools	14 (46.7%)	14 (46.7%)	28 (46.7%)
Cabo de Santo Agostinho – State schools	4 (13.3%)	4 (13.3%)	8 (13.3%)
Jaboatão dos Guararapes – State schools	12 (40.0%)	12 (40.0%)	24 (40.0%)
Number of eligible students per school*	278 (153)	268 (177)	273 (164)
**Number of respondents per school**	37.3 (9.4)	35.2 (9.8)	36.2 (9.6)
**Student characteristics**			
**Number of student respondents**	1,119	1,055	2,174
**Gender (N = 2,146)**			
Female	514 (46.4%)	502 (48.4%)	1,016 (47.3%)
Male	567 (51.1%)	515 (49.7%)	1,082 (50.4%)
Other^†^	28 (2.5%)	20 (1.9%)	48 (2.2%)
**Age (N = 2,174)**	14.5 (1.5)	14.4 (1.5)	14.4 (1.5)
**Planning to study beyond high school (N = 2,045)**			
Yes	830 (79.2%)	765 (76.7%)	1,595 (78.0%)
No	218 (20.8%)	232 (23.3%)	450 (22.0%)
**Missed school in the last month**^**#**^ **(N = 2,070)**			
None	704 (66.3%)	709 (70.3%)	1,413 (68.3%)
1 or 2 days	233 (21.9%)	184 (18.2%)	417 (20.1%)
> 2 days	125 (11.8%)	115 (11.4%)	240 (11.6%)
**Physical violence by parents or figures of authority (N = 1,918)**			
Yes	102 (10.1%)	116 (12.8%)	218 (11.4%)
No	912 (89.9%)	788 (87.2%)	1,700 (88.6%)
**Forced sexual intercourse (N = 1,953)**			
Yes	53 (5.1%)	59 (6.4%)	112 (5.7%)
No	983 (94.9%)	858 (93.6%)	1,841 (94.3%)
**Ever had sexual intercourse for money or a gift (N = 2,054)**			
Yes	49 (4.6%)	65 (6.5%)	114 (5.6%)
No	1,011 (95.4%)	929 (93.5%)	1,940 (94.4%)
**Sexual intercourse for money or a gift in the last 6 months (N = 2,048)**			
Yes	36 (3.4%)	34 (3.4%)	70 (3.4%)
No	1,025 (96.6%)	953 (96.6%)	1,978 (96.6%)
**CKAQ-R score (N = 1,779)**	6.5 (2.1)	6.7 (2.3)	6.6 (2.2)
**Knowledge of risks of online sex score (N = 1,616)**	9.9 (3.2)	10.0 (3.1)	10.0 (3.1)
**Would seek help in cases of online abuse (N = 2,010)**	742 (70.7%)	674 (70.1%)	1,416 (70.4%)
**Participated in sexual abuse prevention activities (N = 2,089)**	277 (25.5%)	283 (28.2%)	560 (26.8%)

Data are n (%) or mean (SD).

CKAQ-R = Children's Knowledge of Abuse Questionnaire – Revised; SD = Standard Deviation

*Total number of students fitting eligibility criteria, as communicated by the school.

#Number of days reported by survey participants

†Others include transgender, non-binary, or those who respond ‘other’ when asked about their gender

Students’ average age was 14 years old, and 50.4% were male. More than one in twenty adolescents reported having experienced the most severe forms of sexual abuse and exploitation, with 5.7% reporting ever having sexual intercourse against their will, while 5.6% reported ever having sexual intercourse in exchange for money, gifts, or favors.

The mean CKAQ-R score was 6.6 at baseline (out of 12 questions), while the mean score for knowledge of risk associated with online sex was 10.0 (out of 15 questions). Around 26% of participants reported having participated in educational activities or classes on preventing or reacting to sexual abuse. All characteristics were well balanced between study arms, except for ever having sexual intercourse in exchange for money, gifts, or favors which was higher in the GWV arm (6.5% vs 4.6%)

One school from the control arm withdrew from the study after the baseline survey.

### Intervention implementation

The intervention was implemented between May and August 2024. Data about the implementation of the intervention are reported in Table 2.

**Table 2:** Intervention implementation (N = 30 schools in GWV arm).

	N (%) Mean (SD)	Endline CKAQ-R	P-value^§^
**School received GWV training**	27 (90%)		
Number of teachers trained*	1.7 (0.9)		
**Number of GWV components implemented** ^**†**^	3.7 (2.7)		
**School implementation level** ^**§**^			
Not implemented or no data ^¶^	10 (33.3%)	7.3 (0.4)	
Lower (≤3 components)	11 (36.6%)	7.6 (0.7)	0.56
Higher (>3 components)	9 (30.0%)	6.9 (0.5)	

*mean (SD) number of teachers recorded on training log. Based on 27 schools that attended training.

†mean (SD) number of GWV components used in class. Based on the teachers’ survey, completed by 32 teachers in 20 schools.

‡Defined according to the number of GWV components used in class. Based on the teachers’ survey, completed by 32 teachers in 20 schools.

§p-value comparing the mean CKAQ-R score between the three implementation levels. Adjusted for strata and baseline CKAQ-R.

¶Two schools (6.7%) did not receive the intervention, and eight (26.6%) received the intervention but did not complete the implementation survey

Among the 30 schools randomised to receive the GWV intervention, 3 (10%) schools enrolled teachers in GWV who did not attend the training. In the remaining 27 schools, an average of 1.7 teachers participated in the GWV training, with a range of 1–4 per school.

Implementation data were derived from a survey conducted with teachers in August 2024, following the GWV training sessions and prior to the endline assessment. The survey was completed by 32 teachers from 20 schools. On average, they reported using 3.7 GWV components in their classes. Four teachers (12%) reported not having implemented any GWV components. The reasons for this included not receiving the materials, difficulty in determining the best strategy to address such a sensitive subject, lack of time, and plans to implement the components in the future.

### Endline and outcomes

A small proportion (2.8%) of parents/guardians of eligible students refused their children’s participation in the survey. The endline survey was conducted in September 2024 and completed by 2,139 students from 59 schools (36.2 students per school on average). Participants’ characteristics are reported in Table 3.

**Table 3:** Endline characteristics.

	Control	GWV	All
**School characteristics**			
**Number of schools**	29	30	59
**Number of respondents per school**	36.2 (9.7)	36.3 (7.4)	36.3 (8.5)
**Student characteristics**			
**Number of student respondents**	1,050	1,089	2,139
**Gender (N = 2,115)**			
Female	517 (49.9%)	531 (49.2%)	1,048 (49.6%)
Male	501 (48.4%)	529 (49.0%)	1,030 (48.7%)
Other*	18 (1.7%)	19 (1.8%)	37 (1.75%)
**Age (N = 2,139)**	14.7 (1.6)	14.9 (1.5)	14.8 (1.5)

Data are n (%) or mean (SD).

*Others include transgender, non-binary, or those who respond ‘other’ when asked about their gender.

The results for the primary and secondary outcomes are reported in Table 4.

**Table 4:** Primary and secondary outcomes.

	Control (n = 1,050)	GWV (n = 1,089)	Comparison*		
	N (%) Mean (SD)	N (%) Mean (SD)	Adjusted Mean difference, RR, or RD	95% CI	p-value
**Primary Outcome**					
CKAQ-R^†^	7.21 (1.8)	7.33 (1.9)	0.02	−0.19 to 0.23	0.87
**Secondary Outcomes**					
Knowledge of risks associated with online sex	11.51 (2.1)	11.50 (2.1)	−0.12	−0.35 to 0.11	0.31
Seeking help in cases of online abuse	858 (81.7%)	870 (79.9%)	RD = −2.5% RR = 0.96	−6.7% to 1.7% 0.91 to 1.02	0.24
Participation in sexual abuse prevention activities	285 (27.1%)	289 (26.5%)	RD = −0.8% RR = 0.93	−5.1% to 3.5% 0.78 to 1.11	0.72

CKAQ-R = Children's Knowledge of Abuse Questionnaire – Revised; CI = Confidence interval; RD = Risk difference; RR = Risk ratio

*All comparisons stratified by strata. For CKAQ-R and knowledge of online risks, analyses also adjusted for school mean baseline CKAQ-R and knowledge of online risks scores, respectively.

†Intracluster correlation coefficient for CKAQ-R = 0.06.

There was no missing data for the primary or secondary outcomes among the 2,139 students who completed the survey. The mean CKAQ-R score at endline was 7.33 in the intervention schools, compared to 7.21 in the control schools, with no statistically significant difference between arms (adjusted mean difference (AMD): 0.02, 95% CI: −0.19 to 0.23, p = 0.87).

There was no statistically significant difference between the two arms for the four secondary outcomes. The knowledge risk score for online sexual exploitation was 11.50 in GWV schools compared to 11.51 in control schools (adjusted mean difference (AMD): −0.12, 95% CI: −0.35 to 0.11, p = 0.31). Additionally, 79.9% of students in GWV schools reported that they would seek help if their intimacy were exposed or if they were sexually harassed online, compared to 81.7% in control schools (risk difference (RD): −2.5%, 95% CI: −6.7% to 1.7%, p = 0.24). Furthermore, 26.5% of students in GWV schools reported having participated in educational activities or classes on preventing or responding to physical sexual abuse, compared to 27.1% in control schools (RD: −0.8%, 95% CI: −5.1% to 3.5%, p = 0.72).

### Interaction

We examined the interaction between students’ gender and age regarding the intervention's effect on the primary outcome. No evidence of interaction with age was found (see Table 5); however, there was borderline evidence of an interaction by gender, showing a greater difference between the arms in CKAQ-R scores for girls than for boys (interaction for boys vs girls: −0.34, 95% CI: −0.65 to −0.03, p = 0.03).

**Table 5:** Interaction analysis by gender and age.

	Control	GWV	Mean difference*	Interaction*	Interaction p-value*
**By gender** ^‡^					
Female (n = 1,053)	7.29	7.56	0.19		
Male (n = 1,034)	7.14	7.08	−0.15	−0.34 (−0.65 to −0.03)	0.032
**By age**					
<15 years (n = 815)	6.86	6.93	0.00		
≥15 years (n = 1,324)	7.43	7.56	0.05	0.05 (−0.31to 0.42)	0.20^†^

Data are mean CKAQ-R at endline

*Comparing GWV arm to the control arm, using a mixed-model, adjusted for strata and school mean baseline CKAQ-R.

†Test for interaction assuming a linear interaction with age.

‡Transgender female or male includes female or male, respectively. Non-binary and ‘other’ are not included in this analysis.

### Dose-response

In a prespecified exploratory analysis, the 30 schools in the GWV arm were classified into three groups based on teachers’ responses to the implementation survey (Table 2). Ten schools (33.3%) either did not implement the intervention or lacked implementation data; 11 schools (30.0%) reported implementing an average of 3 or fewer GWV components; and 9 schools (36.6%) reported having implemented more than 3 components. Supplementary Material 2 details the components implemented by the teachers trained in the GWV methodology. There was no significant difference in adjusted mean CKAQ-R at endline between these 3 groups (p = 0.56, Table 2).In a post-hoc instrumental variable analysis, the causal effect of the intervention on the primary outcome in the 27 schools that receive the intervention was estimated to be 0.02 (AMD) (95% CI: −0.20 to 0.24, p = 0.86).

## Discussion

This trial found no evidence that the Growing Up Without Violence curriculum improved students’ knowledge of risks related to sexual abuse and exploitation. We also did not observe any effect on self-reported knowledge of online sexual risks, intentions to seek help in cases of online abuse, or participation in activities focused on sexual abuse prevention.

While it is difficult to pinpoint the exact reasons for the lack of intervention effects observed in this trial, several factors could contribute to this outcome. These findings may reflect flaws in the GWV's rationale and assumptions (e.g., the belief that changes in knowledge would lead to changes in behaviour), ineffective implementation strategies, or contextual challenges to implementation. Furthermore, the four-month follow-up period may have been insufficient to capture the full effects of the intervention on knowledge retention and behavioural change. Additionally, while the selected outcomes captured knowledge and intention to seek help, they may not reflect other potentially important impacts of the programme, such as actual reporting behaviour, teacher–student relationships, or longer-term shifts in attitudes and social norms.

The intervention's theory of change assumes that improvements in knowledge will translate into protective behaviour and disclosure. This relationship may not hold in all contexts. Structural factors may moderate the effectiveness of knowledge-based prevention programmes. For instance, students’ willingness to report abuse depends not only on their knowledge of what constitutes abuse, but on the availability of accessible and trusted reporting pathways. In Brazil, formal reporting mechanisms exist, including the Disque Denúncia (public hotline) and the Vara da Infância e Juventude (childhood and youth court). However, awareness of, trust in, and practical access to these services among adolescents in high-risk settings cannot be assumed. This trial was a pragmatic trial and aimed to assess the intervention as delivered in practice. While the GWV training is standardised, it is likely that delivery at the school level will vary importantly, and teachers may face barriers to implementation. This reflects the challenges of implementing the intervention, as would happen in practice.

We note that there was some increase between baseline and endline in some of the outcomes. For example, the proportion of students who would seek help in case of online abuse increased by around 10% in both groups. This increase was seen in both groups and not apparently related to the intervention. It could be that CSAE knowledge has improved with the standard curriculum or reflect a “Hawthorne effect” with students being more aware of the survey topic at endline than at baseline. Further analysis of this change over time could be of interest. Item-level analysis of questionnaire responses could provide additional insight into which specific aspects of CSAE knowledge are most amenable to change through school-based interventions and would be a valuable direction for future research.

Furthermore, the moderate level of baseline knowledge on the online sexual risk scale (mean 10.0 out of 15) suggests that students already had substantial prior knowledge before the intervention, which may have reduced the scope for measurable gains — a potential ceiling effect that could partly explain the null result. Although spillover effects between schools cannot be ruled out, schools were geographically dispersed across the two municipalities, and we have not identified mechanism by which spillover would be expected to occur.

Previous studies of school-based prevention of child sexual abuse demonstrated an overall effectiveness of programmes targeting children’s knowledge of risks and protective behaviours [11,36]. Although the findings from these studies are not generalisable to the GWV intervention design or our study context, they indicate that school-based interventions can affect students’ CSAE knowledge levels. Previous findings from a non-published GWV qualitative evaluation in a different Brazilian municipality showed that teachers considered school-based actions on CSAE urgent and valued the GWV content. However, they faced many barriers to implementation, including limited resources and competing priorities for teachers’ time.

Similarly, we hypothesize that the null results from our trial may be partially due to implementation challenges and contextual barriers affecting effectiveness. These include a shortened implementation timeline, low school engagement, high student absenteeism, and obstacles to positive teacher-student relationships. We discuss these factors in more detail below.

### Reduced timeline for implementation

Extended negotiations with municipal and state authorities to finalise agreements with Canal Futura and secure authorization for school-based research activities reduced the implementation timeline from 8 months to just 3 months. This shortened interval between baseline and endline limited opportunities for effective GWV implementation, resulting in a low dose of the intervention being delivered. Additionally, students who received the curriculum had their chances for reflection, abstract conceptualisation, and active experimentation with GWV concepts significantly constrained.

Despite this reduced timeline, our findings show that most teachers (66.6%) reported delivering at least some of the GWV components. Conversely, only 26.5% of students recalled ever participating in CSAE prevention activities. This discrepancy may suggest that, particularly in larger schools, the number of students who have direct contact with trained teachers is limited. Implementing strategies to ensure coverage in all classrooms could help expand the intervention's reach.

### Low school engagement

The reduced timeline of the intervention may have provided insufficient time for teachers to conduct GWV activities alongside the demanding requirements of the Brazilian national curriculum. Low engagement could also stem from cultural and religious barriers to the intervention's content. For instance, one school withdrew from the intervention, deeming the survey questions on sex and violence inappropriate for children and citing parental opposition. Resistance to GWV might also relate to teachers feeling uncomfortable with the content or holding attitudes that contradict the intervention's objectives. However, lack of engagement alone cannot fully account for the trial's findings, as we did not observe improved outcomes in schools with higher implementation levels, nor did we see an increase in students recalling exposure to CSAE activities in intervention schools compared to control schools.

### Students’ high absenteeism

High levels of absenteeism may have adversely affected students’ engagement with the intervention, resulting in a low dose of exposure. During both baseline and endline fieldwork, our teams found that nearly a third of the sampled students were absent from school. Additionally, baseline findings indicated that one in five students reported interrupting their studies for more than a month for reasons unrelated to the COVID-19 pandemic. This high absenteeism reduces children's likelihood of exposure to GWV activities, which, in turn, affects their recall of intervention activities and content learning.

Moreover, research indicates that school absenteeism is linked to increased youth vulnerability, including higher rates of potentially harmful sexual behaviours, psychiatric disorders, externalizing behaviours, youth crime, and alcohol and substance misuse [37]. Several of these risks associated with absenteeism overlap with risks of sexual exploitation, suggesting that some of the students who would benefit most from interventions like GWV are also more likely to miss school. Furthermore, students who are frequently absent are also less likely to be captured in the survey sample itself, meaning our findings may skew toward more engaged students and underrepresent those who are most vulnerable, the very group the intervention aims to reach.

### Barriers impeding positive teacher-student relationships

Pedagogical research indicates that a positive educational environment is central to effective learning, critical thinking, and students’ development [38]. Findings from our baseline survey revealed that a high proportion of students felt they were not often allowed to express their opinions (46.0%) or ask questions in the classroom (43.0%). Additionally, more than two-fifths of surveyed students believed their peers frequently disrupted classes, which may have hindered teachers’ ability to deliver content effectively.

While most students perceived their teachers as motivated (80.5%), the majority reported that teachers rarely engaged with them (70.6%) and showed no interest in their lives outside school (57.5%). These findings indicate a lack of equitable dialogue and engagement between some students and their teachers, suggesting that students may not feel they have a voice in their relationships with their educators. This could undermine trust and engagement in CSAE prevention activities addressing particularly sensitive topics.

In interventions aimed at promoting normative change, the absence of space for dialogue and open discussion may serve as a significant barrier [39]. Furthermore, if students’ perceptions are accurate and some teachers demonstrate a lack of interest in students’ out-of-school experiences, these teachers may be less likely to engage meaningfully with the intervention topics or proactively identify students in need of support. Lastly, students’ disruptive behaviour could further impede teachers’ ability to deliver GWV content effectively.

### Strengths and limitations

To our knowledge, ours was the first rigorous evaluation of a school-based intervention to prevent CSAE in Brazil. The analysis plan was pre-specified, with a limited number of comparisons. Furthermore, all the data and analysis had been prepared before unmasking, limiting the risk of chance finding.

The study, however, had several limitations, outlined below.

#### Generalisability.

We conducted the study in two municipalities in Pernambuco State, in the Northeast of Brazil. Pernambuco State is where the research sponsor, the Freedom Fund, has a hotspot for CSAE intervention. Canal Futura selected Cabo and Jaboatão as implementation sites because they had good connections with the local authorities and had not implemented GWV there recently. Although we have no reason to believe the trial results would differ in other regions of the country, we cannot be sure they are generalisable.

#### Challenges to implement GWV as planned.

Delays in obtaining authorization from local authorities significantly shortened the timeframe between baseline and endline assessments. Consequently, the trial was limited to a restricted implementation period for GWV activities, capturing only 3 of the originally planned 8 months. Given the sensitive nature of the topic and the deeply ingrained social norms that support or excuse CSAE in the research context, it is likely that the GWV implementation period was insufficient to yield meaningful results. Additionally, there is insufficient data to evaluate the quality or consistency of implementation, particularly given the intervention's unstructured nature. We conducted an initial online survey with all teachers enrolled in the intervention, but low participation (40%) reduced the utility of these data. Furthermore, the dose-response analysis relied on the number of components implemented as a proxy for dose, which does not capture variation in the specific components delivered or the quality of implementation.

Additionally, we have no systematic data on teacher absenteeism or turnover during the study period. High teacher turnover or absences could have reduced continuity of delivery, as schools that lost their trained teachers’ mid-intervention would have had limited capacity to maintain implementation.

#### Statistical power and analysis.

Although the number of respondents was lower than anticipated due to absenteeism, the study enrolled a substantial number of schools and maintained sufficient power to detect a moderate to large effect size. With an intraclass correlation coefficient (ICC) of 0.06 and an average cluster size of 36 students per school, the power to detect an effect size of 0.25 remained above 90%. The confidence interval for the primary outcome rules out any significant effect, although we cannot entirely rule out a small benefit. We also note that one school dropped from the study after randomisation. This could have biased the results (making the two randomised groups less comparable), if this decision to withdraw was influenced by the randomisation outcome. This cannot be ruled out but appears unlikely given the school withdrew early on as it felt that the sensitive nature of the research topic would not be appropriate for its students, but it was actually allocated to the control arm.

#### Cross-sectional design.

As the trial used independent samples of students at baseline and endline, individual-level change in knowledge or behaviour could not be measured. This means we cannot determine whether students who were directly exposed to the GWV curriculum improved their knowledge over time. This is an inherent limitation of the repeated cross-sectional design, though — as noted above — this approach was adopted deliberately given the high levels of absenteeism observed and the school-level nature of the intervention.

#### Dose-response analysis.

We note that the dose-response analysis used the number of GWV components delivered as a proxy for implementation dose, rather than examining which specific components were delivered or the quality of their delivery. While this provides a useful summary measure, it does not capture variation in the content or fidelity of implementation across schools.

## Conclusion

This study did not find an impact of GWV on the intended outcomes. Several factors may have contributed to this result: the intervention might have been implemented with insufficient fidelity or intensity to produce measurable change, the true effect of GWV could be small and fall below the study's detectable threshold, or the intervention may simply lack effectiveness. Based on our findings, we cannot draw definitive conclusions about the intervention's potential to effect change.

Future impact evaluations of GWV and similar programmes should ensure adequate investment in comprehensive process evaluations, including systematic monitoring of implementation quality, fidelity, and dose, as well as broader outcome measurement beyond knowledge scores.

Implementation could be strengthened by training multiple educators per school or employing training-of-trainers approaches to reinforce the content and broaden outreach, and by ensuring sufficient time is allocated for delivery before outcome assessment.

CSAE is a pervasive issue that has devastating effects on children's health, well-being, and development. Previous evaluations of school-based CSAE prevention programs have yielded mixed results. To ensure that resources are allocated to effective strategies, further rigorous evaluations and implementation studies are essential.

## Supporting information

S1 Table Summary of the GWV intervention's components, themes, targeted audience, and content. (ODT)

S2 Table Growing up Without Violence (GWV): Frequency of each component implementation (n = 32). (DOCX)
