# Supplementary material for: Growing up without violence (GWV): Results of a cluster randomised trial of a school-based intervention preventing adolescent sexual abuse and exploitation in Brazil
Source: PLoS One. 2026 Jul 30;21(7):e0342274. doi: 10.1371/journal.pone.0342274 (PMC13422835; doi:10.1371/journal.pone.0342274)
Supplement: S2 Table — (DOCX) [file pone.0342274.s002.docx]

**S2 Table**. Growing up Without Violence (GWV): Frequency of each component implementation (n=32)

| **Implementation components** | **Frequency n(%)** |
| --- | --- |
| GWV Educational material (hard copies) | 9 (28.1%) |
| Training guidance | 9 (28.1%) |
| Pen drive with educational videos | 13 (40.6%) |
| Educational material on sexual exploitation | 6 (18.8%) |
| Video on the body awareness | 14 (43.8%) |
| Video on sexual abuse | 13 (40.6%) |
| Video on sexual exploitation | 11 (34.4%) |
| Magazine on rights of children | 3 (9.4%) |
| Booklet on prevention of sexual abuse and exploitation | 9 (28.1%) |
| Booklet on sexual violence against children and adolescents | 7 (21.9%) |
| Online material on prevention of child sexual abuse and exploitation | 4 (12.5%) |
| Case studies | 11 (34.4%) |
| Material on children’s rights and legislation | 7 (21.9%) |
| Campaign material on domestic sexual violence against children | 3 (9.4%) |
| Unable to implement any component | 3 (9.4%) |
| No response | 1 (3.1%) |
