# Supplementary material for: Growing up without violence (GWV): Results of a cluster randomised trial of a school-based intervention preventing adolescent sexual abuse and exploitation in Brazil
Source: PLoS One. 2026 Jul 30;21(7):e0342274. doi: 10.1371/journal.pone.0342274 (PMC13422835; doi:10.1371/journal.pone.0342274)
Supplement: Supplementary file 3 [file pone.0342274.s001.odt]

| S1 Table. Summary of the GWV intervention's components, themes, targeted audience, and content. |
| --- |
| Component | Themes | Target audience | Content |
| Sexual Abuse | E1: Unmasking abuse | Children and educators | Reporting sexual abuse to the competent authorities and social services Challenging the misconception that abuse is limited to certain locations or cultures Learning the difference between abuse and sexual exploitation |
| E2: Is it just affection? | Children | Learning the difference between sexual abuse and caring behaviours from adults, and how to spot sexual abuse when you’re a child Challenging the misconception that abusers are often strangers |
| E3: Danger at home | Educators | Challenging the idea that sexual abuse does not take place in conventional/biological families Breaking abuse cycles that may lead abused children to be sexually abusive towards other children when they grow up |
| E4: Where there’s smoke… | Educators | Learning how to spot signs of abuse in children, such as changes in behaviours, differences in school marks, how children act when talking about sex, etc. Reinforcing the role of educators in spotting abuse and reporting it |
| E5: It is necessary to listen | Educators and children | Recognising the need to listen to children and talk to them as crucial in reporting any abuse Challenging the notion that children often lie about abuse Learning about who are the competent authorities to report abuse |
| E6: Collaborating to protect | Educators and children | Recognising the need to work together and in collaboration with social services to protect children Learning about how to report and different ways of reporting |
| E7: Wolf dressed as lamb | Children | Challenging the idea that abusers are usually unknown to the victim Recognising that abuse can happen in relationships with people that children trust Discussing that abusers may also need psychological assistance, as they might have been victims of abuse Recognising the need to break sexist cultural stereotypes to protect children and ensure that boys report sexual violence incidents, and recognising that women can also be abusers |
| E8: ways to reporting | Children and educators | Recognising the importance of reporting Learning about what do different social services do and the existence of difference reporting channels Learning how children can report |
| Sexual exploitation | E1: Breaking labels | Children | Challenging the misconception that boys can’t be victims of sexual exploitation Learning about the family and social pathways that lead boys and girls into sexual exploitation Learning about grooming techniques Learning about the importance of reporting and which authorities they should contact |
| E2: Virtual Danger | Children | Learning about risks associated with online sexual exploitation Learning how to recognize online grooming techniques Learning how children can report online sexual crimes |
| E3:Tourism | Children | Learning about family and social pathways that lead girls into sexual exploitation in tourism Understanding the need to report sexual exploitation to the competent authorities |
| E4: hotel networks | Children | Challenging the misconception that adults are “helping” children by purchasing sexual services from them Understanding the need to report sexual exploitation incidents to the competent authorities |
| E5: Reporting helps others | Children | Learning how to recognize a situation of sexual exploitation Learning about the family and social pathways that lead children into sexual exploitation Challenging the idea that poverty can justify sexual exploitation of children |
| Body | E1: My body, my rules | Children | Learning when is right and wrong to have adults touching your intimate parts |
| E2: Private and Public | Children | Learning the concept of privacy and when it’s right and wrong to be naked, and around who |
| E3: The right to say no | Children | Learning that you have the right to refuse a certain social interaction with someone |
| E4: Love and abusive relationships | Children | Understanding abusive relationships and toxic masculinity |
| E5: Gender stereotypes | Children | Learning about homophobia and gender stereotypes and how gender expression should not be associated with sexual orientation (homosexual men aren’t necessarily more feminine, and the other way around) Nurturing healthy relationships of acceptance between friends |
| E6: Internet and the media | Children | Learning about the dangers associated with meeting and dating people online Understanding the dangers associated with “leaking nudes”/ sending nudes |
| E7: Online emotional health and self-harm | Children | Learning about Cyberbullying |
| E8: Toxic masculinity | Children | Learning about toxic masculinity, peer pressure and homophobia |
| E9: My body, my rules | Children | Learning that you should only have sexual interactions when you want to Learning about contraception |
| E10: Grooming and self-protection | Children | Learning about online grooming and how gender and race intersect and lead to an increased vulnerability of black women Showcasing online helplines that children can use to report inappropriate online interactions |
| E11: Digital reputation | Children | Understanding the dangers associated with “leaking nudes”/ sending nudes Learning how you can help someone who is currently going through a mental health crisis due to a leaked nude |
| E12: Online relationships | Children | How to safely meet people online |
